# A Scalable Protocol for Ex Vivo Production of CAR-Engineered Human NK Cells

**DOI:** 10.3390/mps8050102

**Published:** 2025-09-05

**Authors:** Supreet Khanal, Nirjal Bhattarai

**Affiliations:** Tumor Vaccine and Biotechnology Branch, Division of Cell Therapy II, Office of Cellular Therapy and Human Tissues, Office of Therapeutic Products, Center for Biologics Evaluation and Research, U.S. FDA, Silver Spring, MD 20993, USA; supreet.khanal@fda.hhs.gov

**Keywords:** chimeric antigen receptor, CAR-NK, cell manufacturing, expansion, G-Rex

## Abstract

Chimeric antigen receptor-expressing NK (CAR-NK) cells represent an advancing frontier in cancer immunotherapy, building upon decades of natural killer cell research and recent breakthroughs in CAR technology. While early CAR-NK manufacturing protocols have demonstrated feasibility, existing manufacturing methods, whether utilizing cord blood or peripheral blood sources, often require extended culture periods and intensive labor, creating bottlenecks for widespread therapeutic application. To address these manufacturing hurdles, we have developed an optimized protocol for ex vivo CAR-NK cell production from human peripheral blood that incorporates lessons learned from previous methodologies while introducing novel efficiency improvements. This protocol offers a practical solution for scalable CAR-NK cell manufacturing that can be readily adapted across different production facilities, potentially accelerating the clinical development of CAR-NK therapies.

## 1. Introduction

Chimeric antigen receptor (CAR)-expressing natural killer (NK) cells represent a promising cellular immunotherapy approach to a wide variety of human diseases [1,2]. As a key player of the innate immune system, NK cells can rapidly recognize and kill target cells in a non-specific manner, independently of the antigen presentation, making them an ideal option for developing allogeneic, off-the-shelf cellular therapies. The ex vivo manufacturing of CAR-NK cells requires NK cells’ isolation, genetic manipulation, and cellular expansion under conditions that do not affect its quality (e.g., viability, purity etc.), safety, and potency [3,4,5]. Human NK cells obtained from peripheral blood mononuclear cells (PBMCs), umbilical cord blood (UCB), induced pluripotent stem cells (iPSCs), or the NK-92 cell line have been used to manufacture CAR-NK cells [6,7,8,9]. CAR-NK cells manufactured using NK cells from various sources have demonstrated clinical efficacy, and their application has expanded beyond oncology to non-oncology indications such as autoimmune diseases [1,10,11,12,13]. Among various NK cell sources, peripheral blood-derived NK cells are more readily available, making them a widely used cell source for manufacturing CAR-NK cells [2,6,14].

To manufacture CAR-NK cells, NK cells are first isolated from peripheral blood. Generally, immunomagnetic bead-based selection methods are used to achieve high cell purity. Good isolation techniques are also crucial for achieving high purity in the final CAR-NK cell product. Generally, obtaining over 90% pure NK cells is recommended, as it leads to obtaining highly pure CAR-NK cell products with little to no contamination from non-NK cells [15]. Once highly pure NK cells are obtained, vector-mediated CAR gene transfer into NK cells is achieved. Usually, viral vectors are used for this process, but non-viral vectors may be also used. Following CAR gene transfer and CAR expression, NK cells are expanded in culture. One of the challenges during CAR-NK cell manufacturing is achieving robust cellular expansion while maintaining functionality. Traditional culture systems are often constrained by low cellular yields and poor viability at high densities. The G-Rex (Gas-permeable Rapid Expansion) system effectively addresses these limitations by enabling high-density, large-volume cultures with enhanced gas exchange. This scalable and efficient system provides a solution to achieve high NK cell expansion during ex vivo culture [10,16,17,18,19].

In this protocol, we present a detailed step-by-step methodology for ex vivo manufacturing of CAR-NK cells using primary NK cells from human peripheral blood using a G-Rex system.

## 2. Experimental Design

**Figure 1:**
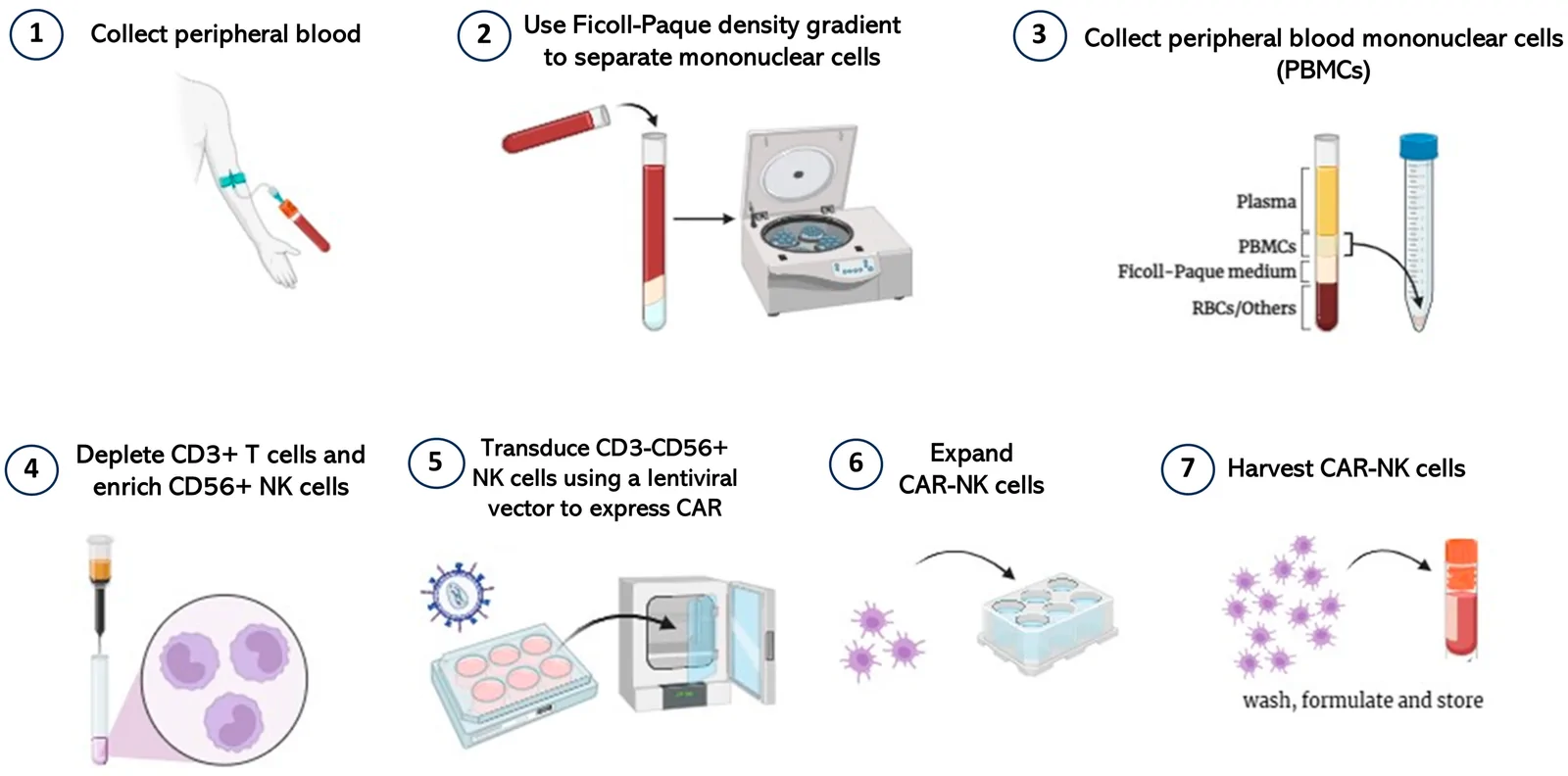
Schematic representation of the steps involved in the ex vivo manufacturing of human CAR-NK cells using peripheral blood: The CAR-NK cell manufacturing process begins with (**1**) the collection of human blood and (**2**) the isolation of peripheral blood mononuclear cells (PBMCs) using a Ficoll–Paque density gradient. (**3**) The collected mononuclear cells undergo (**4**) CD3^+^ T cell depletion and CD56^+^ NK cell isolation. (**5**) The purified NK cells are then activated and transduced with a lentiviral vector to express the CAR transgene. (**6**) Transduced CAR-NK cells are expanded for up to 14 days, (**7**) then harvested, washed, formulated, and stored.

This protocol provides a detailed step by step process for the isolation of human PBMCs from whole blood or buffy coat, purification of human NK cells, lentiviral vector-mediated transduction of NK cells for CAR expression, and expansion of transduced CAR-NK cells in the G-Rex system (Figure 1, Table 1).

## 3. Procedure

### 3.1. Cell Collection

#### 3.1.1. Isolation of Peripheral Blood Mononuclear Cells (PBMCs) from Buffy Coat or Whole Blood (Processing Time: 60–90 min)

1. Dilute whole blood with sterile PBS at a 1:1 ratio (e.g., 10 mL blood + 10 mL PBS). For buffy coat, dilute with sterile PBS at a 1:2 or 1:3 ratio (e.g., 10 mL buffy coat + 20 mL or 30 mL PBS) depending on the viscosity observed with buffy coat. Use fresh whole blood or buffy coat (obtained within 24 h), when possible.
2. Prepare Ficoll–Paque gradient by gently adding 15 mL of Ficoll-Paque to the bottom of a 50 mL conical tube.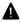 **CRITICAL STEP**: Gently add the diluted whole blood or buffy coat on the top of the Ficoll–Paque gradient very slowly without disturbing the gradient (tip: tilt the tube and slowly dispense the diluted blood or buffy coat from one side of the tube into the gradient).
4. Add up to 25 mL of diluted whole blood or buffy coat per 15 mL of Ficoll–Paque gradient. Add sterile PBS to bring the final volume to 50 mL.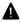 **CRITICAL STEP**: Centrifuge at 800× *g* for 20 min at room temperature. Perform the spin with medium acceleration and no brakes for deceleration to avoid disturbing the density gradient.
6. After centrifugation, four layers will be visible: Plasma (first); white, a cloudy layer containing PBMCs between the plasma/Ficoll interface (second); Ficoll–Paque medium (third); and a cellular pellet containing the red blood cells (RBCs) and other cells such as granulocytes (fourth) (Figure 1, Step 3).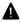 **CRITICAL STEP**: Aspirate and discard the fraction containing plasma. Remove as much plasma as possible without disturbing the PBMC “white cloudy” layer. This helps to prevent contamination of PBMCs with platelets.
8. After discarding the plasma fraction, carefully aspirate the PBMC layer without disturbing the Ficoll–Paque gradient using a sterile pipette and transfer to a new tube. Discard the tube containing the Ficoll–Paque gradient and RBCs.
9. Wash the PBMCs by resuspending in 20 mL of PBS and centrifuge at 300× *g* for 10 min with full acceleration and braking.
10. Discard the supernatant and repeat the wash steps two more times to remove any residual Ficoll gradient and plasma (note: proper washing ensures the removal of platelets and residual Ficoll, which can interfere with downstream applications).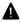 **CRITICAL STEP**: After completion of the wash steps, the cell pellet should appear white in color. If contamination with RBCs is observed, add 10 times the volume equivalent of the RBC lysis buffer (note: if 10 mL of blood or buffy coat was used as a starting material for PBMC isolation, add 100 mL of RBC lysis buffer). Incubate for 5 min at room temperature to lyse RBCs. Following RBC lysis, wash one time with 20 mL PBS as described above.
12. Resuspend the PBMC pellet in complete RPMI media (RPMI 1640 + 1% Pen/Strep + 1% glutamine + 10% heat-inactivated FBS) (tip: use the same volume of complete RPMI to resuspend the PBMC pellet as the initial volume of blood or buffy coat used in Step 1).
13. Count the cells using an appropriate cell counting method (e.g., automated cell counter) to assess viability and cell concentration. Adjust the PBMC concentration to 1 × 10^7^ cells/mL using the complete RPMI media.
14. Label the cells as PBMCs.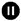 **PAUSE STEP**: PBMCs can be either used immediately for CAR-NK cell manufacturing or cryopreserved for future use. For cryopreservation, resuspend PBMCs in freezing media at a concentration of 1 × 10^7^ cells/mL and aliquot in cryovials. Label the cryovials using cryogenic labels and place in a freezing container (e.g., Mr. Frosty) at −80 °C for 24 h. PBMCs may be stored at −80 °C for the short term (e.g., less than 1 week). For long-term storage, move the cryovials from −80 °C to LN2.

#### 3.1.2. Isolation of NK Cells from PBMCs by CD3 Depletion and CD56 Enrichment (Processing Time: 60–90 min) 

Human NK cells are typically defined as CD3^−^CD56^+^ lymphocytes. To isolate human NK cells from PBMCs, a two-step process is generally used: first CD3^+^ T cells are depleted; second, CD56^+^ NK cells are isolated.

1. For cryopreserved PBMCs, go to Step 2. For fresh PBMCs, go to Step 3.
2. Obtain the required number of cryovials containing cryopreserved PBMCs from storage. Thaw the cells using an appropriate method (e.g., 37 °C water bath). After complete thawing, wash the cells once in PBS (10 mL per 10^7^ cells) and resuspend in 100 µL of MACS buffer per 10^7^ cells.
3. Add 20 µL of anti-CD3 microbeads per 10^7^ cells to the PBMC suspension (note: any commercial NK isolation kits can be used, following the manufacturer’s instructions).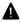 **CRITICAL STEP**: Mix the bead and cell suspension gently using pipette and incubate for 10 min on ice or at 4 °C. Ensure slow and uniform mixing of the beads and cells. Strictly follow the manufacturer’s incubation times and keep cells cold to prevent non-specific binding.
5. Wash the cells by adding 1 mL of MACS buffer per 10^7^ cells.
6. Centrifuge at 300× *g* for 10 min. Aspirate the supernatant completely.
7. Resuspend up to 10 × 10^7^ cells in 500 µL of MACS buffer.
8. To isolate cells using the magnetic separator, either use an MS (for up to 2 × 10^8^ cells) or LS column (for up to 2 × 10^9^ cells).
9. Rinse the column with MACS buffer (500 µL for the MS column or 3 mL for the LS column).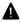 **CRITICAL STEP**: Apply the cell suspension to the rinsed column slowly to maintain column integrity and prevent clogging.
11. Collect the CD3^−^ flow-through fraction, which contains NK cells and other CD3^−^ cells.
12. Wash the column with MACS buffer (500 µL for the MS column or 3 mL for the LS column) collecting all flow-throughs.
13. Discard the column containing CD3^+^ cells unless needed for any other purpose.
14. To isolate the NK cells from the CD3^−^ fraction, count the cells and adjust the cell concentration to 1 × 10^7^ cells/100 µL of MACS buffer.
15. Add 20 µL of anti-CD56 microbeads per 10^7^ cells.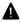 **CRITICAL STEP**: Mix the bead and cell suspension gently using a pipette and incubate for 10 min on ice or at 4 °C. Ensure slow and uniform mixing of the beads and cells. Strictly follow the manufacturer’s incubation times and keep cells cold to prevent non-specific binding.
17. Wash the cells by adding 1 mL of MACS buffer per 10^7^ cells.
18. Centrifuge at 300× *g* for 10 min.
19. Resuspend the cells in MACS buffer. Use 1 mL MACS buffer per 1 × 10^7^ cells.
20. Select an appropriate MACS column as in Step 8. Rinse the column with MACS buffer (500 µL for an MS column or 3 mL for an LS column).
21. Apply the cell suspension to the MACS column and collect the flow-through as the CD3^−^CD56^−^ fraction. Discard the flow-through (CD3^−^CD56^−^ fraction) unless needed for any other purpose.
22. Wash the column with MACS buffer (500 µL for the MS column or 3 mL for the LS column).
23. To collect CD56^+^ NK cells from the column, remove the column from the magnet and add 1 mL of MACS buffer per 10^7^ cells into the column. Using a plunger, flush the cells out of the column into a clean 15 mL conical tube. Label the tube as CD3^−^CD56^+^ NK cells.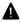 **CRITICAL STEP**: Count the cells using an appropriate cell counting method (e.g., automated cell counter) to assess viability and cell concentration.
25. Assess isolated NK cell purity by flow cytometry after staining with anti-CD3 and anti-CD56 antibodies. Note: We recommend obtaining ≥ 90% CD3^−^CD56^+^ NK cells to minimize T cell contamination in CAR-NK cell product.

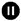
 ** PAUSE STEP:** Isolated NK cells can be either used immediately for CAR-NK cell manufacturing or cryopreserved for future use. For cryopreservation, resuspend the NK cells in freezing media at a concentration of 1 × 10^7^ cells/mL and aliquot in cryovials. Label the cryovials using cryogenic labels and place in a freezing container (e.g., Mr. Frosty) at −80 °C for 24 h. PBMCs may be stored at −80 °C for the short term (e.g., less than 1 week). For long-term storage, move the cryovials from −80 °C to LN2.

### 3.2. NK Cell Activation and Transduction by a Lentiviral Vector for CAR Expression (Processing Time: 24–48 h)

Activation of NK cells prior to lentiviral vector transduction greatly improves lentiviral vector-mediated gene transfer efficiency, resulting in higher CAR protein expression in NK cells. Generally, NK cells are activated using irradiated feeder cells (e.g., K562) in the presence of IL-2 family cytokines; however, feeder-cell-free methods of activation are also used to manufacture CAR-NK cells [17,20]. Following activation, NK cells are transduced with a replication-incompetent lentiviral vector carrying the CAR transgene. The following steps can be followed for NK cell activation and lentiviral vector transduction of NK cells for CAR expression.

1. Wash the isolated fresh NK cells with PBS and resuspend in complete RPMI media in the presence of IL-2 (200–500 IU/mL). If cryopreserved NK cells are used, obtain the required number of cryovials containing cryopreserved NK cells from storage. Thaw the cells using an appropriate method (e.g., 37 °C water bath). After complete thawing, resuspend and wash the cells in 10 mL PBS and resuspend in complete RPMI media.
2. Count the cells using an appropriate cell counting method (e.g., automated cell counter) to assess viability and cell concentration. Adjust the NK cell concentration to 5 × 10^6^ cells per mL.
3. To activate NK cells using feeder cells, co-culture NK cells (5 × 10^6^ cells) with irradiated, replication-incompetent K562 cells at a 1:1 ratio for 24 h.
4. Alternatively, to activate NK cells independent of feeder cells, add PMA (50 ng/mL) and Ionomycin (1 µg/mL) to NK cells (5 × 10^6^ cells) for 24 h.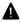 **CRITICAL STEP**: Coat the non-tissue culture-treated plate (6 or 12 well) with retronectin (10 µg/mL in PBS). Add retronectin (0.5 mL) into each well of a 24-well plate or 2 mL into each well of a 6-well plate (note: retronectin coating improves the lentiviral vector transduction efficiency of NK cells).
6. Incubate for 2 h at room temperature or overnight at 4 °C.
7. Wash the retronectin-coated plate with 1 mL PBS and add 1 mL of Superblock solution or 2% BSA in PBS for 30 min at room temperature.

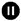
 **PAUSE STEP:** The retronectin-coated plate can be sealed with parafilm and kept at 4 °C for up to 2 weeks (note: preparing the retronectin-coated plate in advance ensures the plate’s availability for the transduction step).

8. Wash the retronectin-coated plate two times with 1 mL PBS before use.
9. Add the lentiviral vector and activated NK cells in the retronectin-coated wells at a multiplicity of infection (MOI) of 20 (note: we recommend optimizing the MOI using primary NK cells or a NK cell line to achieve the desired level of CAR gene expression before executing experiments).
10. Centrifuge the retronectin-coated plate containing the lentiviral vector and NK cells at 1800× *g* for 60 min at room temperature.
11. After centrifugation, incubate the plate at 37 °C and 5% CO_2_.
12. After 12–24 h, wash the cells once using PBS and count the cells using an appropriate cell counting method (e.g., automated cell counter) to assess viability and cell concentration.
13. Resuspend transduced cells in complete RPMI media supplemented with IL-2 (200–500 IU/mL), IL-15 (5 ng/mL), and IL-21 (25 ng/mL) at 0.5–1.0 × 10^6^ cells per mL. Culture transduced cells in a tissue culture plate (e.g., 12-well or 6-well).
14. Incubate the cells at 37 °C and 5% CO_2_ for 48 h.
15.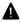 **CRITICAL STEP**: Assess CAR protein expression using an appropriate reagent (e.g., protein L, anti-Fab etc.) using flow cytometry.
16. If CAR expression is acceptable, proceed with CAR-NK expansion.

### 3.3. Expansion of Transduced CAR-NK Cells (Processing Time: 10–14 Days)

The expansion of transduced NK cells is a critical step during CAR-NK cell manufacturing. Generally, the IL-2 family of cytokines (e.g., IL-2, IL-15, IL-21) is commonly used to expand CAR-NK cells ex vivo. The concentration of cytokines used here for CAR-NK cell expansion is comparable with other studies [15,21,22,23]. The concentration of cytokines should be carefully evaluated, as high levels and continuous exposure of cytokines can negatively affect NK cell products [24,25].

1. Wash the transduced CAR-NK cells using 10 mL PBS and resuspend in 10 mL NK cell expansion media containing all three cytokines (IL-2, IL-15, and IL-21).
2. Transfer the cells into an appropriate system for cellular expansion. We recommend using the G-Rex system for CAR-NK cell expansion using NK cell expansion media (note: resuspend 5 × 10^6^ CAR-NK cells in 10 mL expansion media per well of a G-Rex 6-well plate). Maintain CAR-NK cell density between 0.5 and 1.0 × 10^6^ cells per mL and incubate at 37 °C and 5% CO_2_.
3. Monitor cell growth every other day and change the media as needed to maintain cell density (note: to change the media, gently remove the top 80% of the media without disturbing the cells settled at the bottom and add the required amount of fresh NK cell expansion media).
4. After 10 days of expansion, count cells, and if the desired cell number is achieved, prepare the cells for harvest. If additional cell expansion is needed, maintain the cell culture for additional days up to Day 14.
5. Before harvesting CAR-NK cells, assess their total cell number, viability, and CAR protein expression. To harvest cells, gently collect CAR-NK cells from G-Rex to a sterile 50 mL conical tube. Centrifuge at 300× *g* for 10 min. Wash the cells twice with sterile PBS.

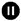
 **PAUSE STEP:** CAR-NK cells can be used immediately for experiments or cryopreserved for future use. For cryopreservation, resuspend CAR-NK cells in freezing media at a concentration of 1 × 10^7^ cells/mL and aliquot in cryovials. Label the cryovials using cryogenic labels and place in freezing container (e.g., Mr. Frosty) at −80 °C for 24 h. PBMCs may be stored at −80 °C for the short term (e.g., less than 1 week). For long-term storage, move the cryovials from −80 °C to LN2.

6. Resuspend the CAR-NK cells in an appropriate NK cell media for experiments. Before using CAR-NK cells for experiments, perform additional testing or characterization as needed (e.g., assess activation and exhaustion markers, the presence of non-NK cellular impurities, sterility, and endotoxin testing, etc.).

## 4. Expected Results

### 4.1. PBMC Isolation

**Figure 2:**
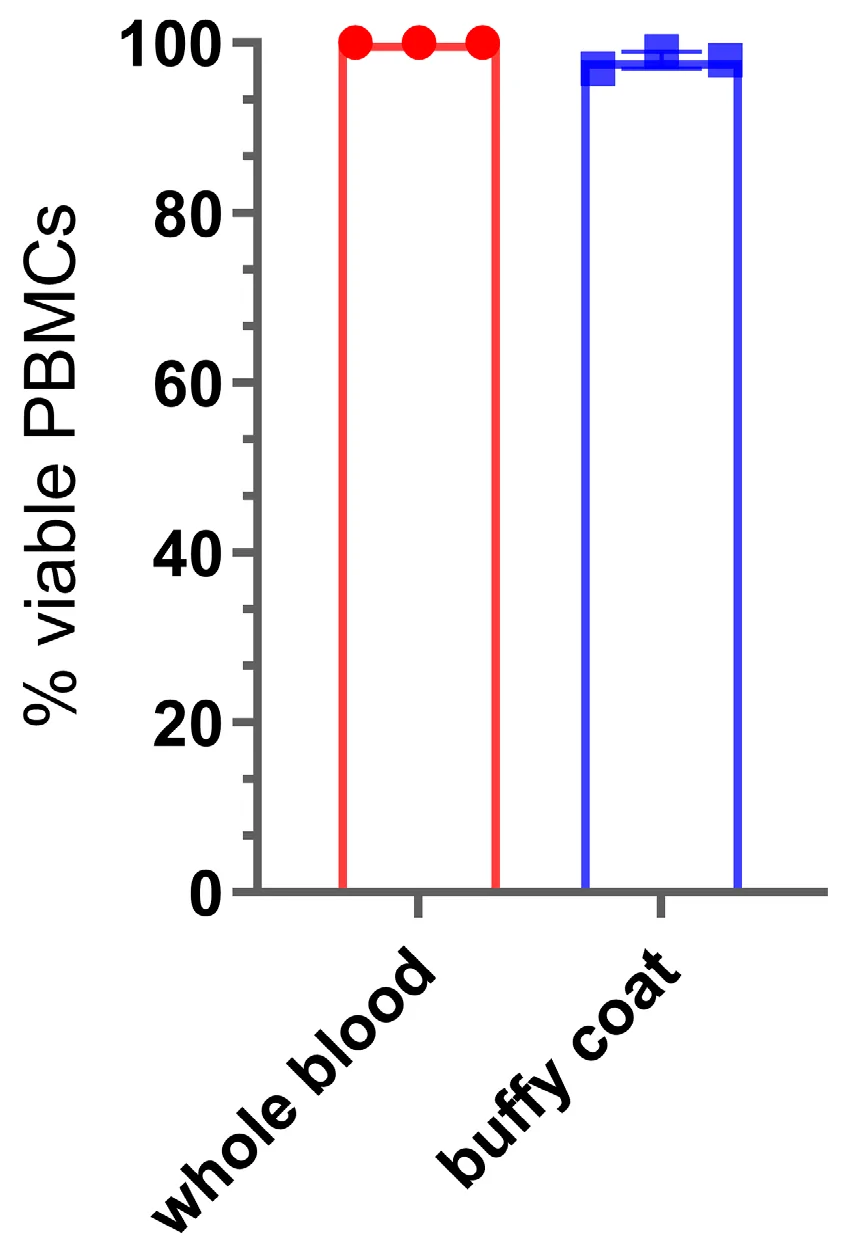
Viability after isolation of PBMCs. Viability was assessed after isolation of peripheral blood mononuclear cells (PBMCs) from whole blood or buffy coat using a Ficoll–Paque density gradient. Each dot represents a donor.

Peripheral blood mononuclear cells (PBMCs) were isolated from whole blood or buffy coat obtained from human donors using Ficoll–Paque gradient centrifugation. Following isolation of PBMCs, the cells were washed, and viability was measured using Trypan Blue and an automated cell counter (Countess II, ThermoFisher). PBMCs obtained from both whole blood and buffy coat had a very high cellular viability of >95% (Figure 2).

### 4.2. NK Cell Isolation

**Figure 3:**
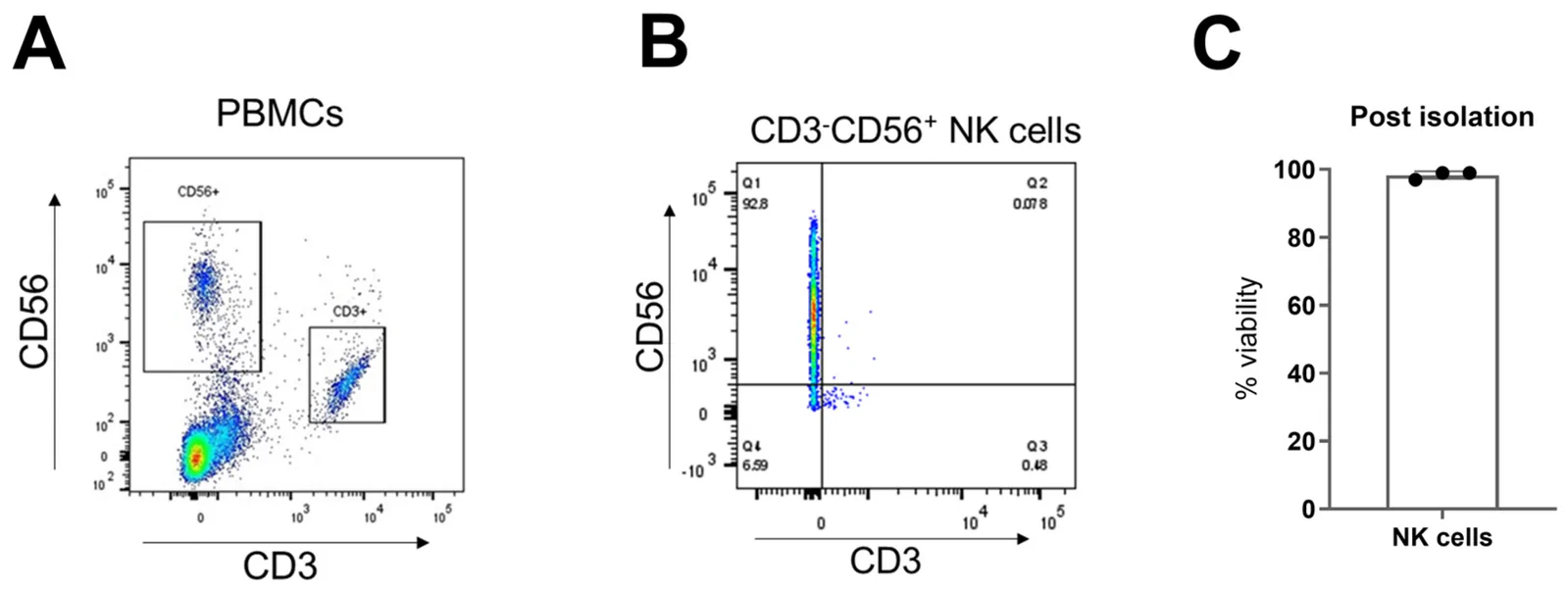
Cell purity before and after NK cell isolation. Cell purity was assessed by flow cytometry before and after the isolation of NK cells from peripheral blood mononuclear cells (PBMCs). Representative flow cytometry plots demonstrate CD56^+^ NK and CD3^+^ T cell populations in (**A**) PBMCs (pre-isolation) and (**B**) NK cells (post-isolation). (**C**) Viability of NK cells post-isolation. Each dot represents a donor.

CD3^+^ T cells and CD56^+^ NK cells were assessed in PBMCs and after NK cell isolation. In PBMCs, both CD3^+^ T cells and CD56^+^ NK cells were present; however, after NK cell isolation, highly pure NK cells (>90%) were obtained (Figure 3A,B). The viability of isolated NK cells was also very high (>95%) (Figure 3C).

### 4.3. NK Cell Activation and Transduction

**Figure 4:**
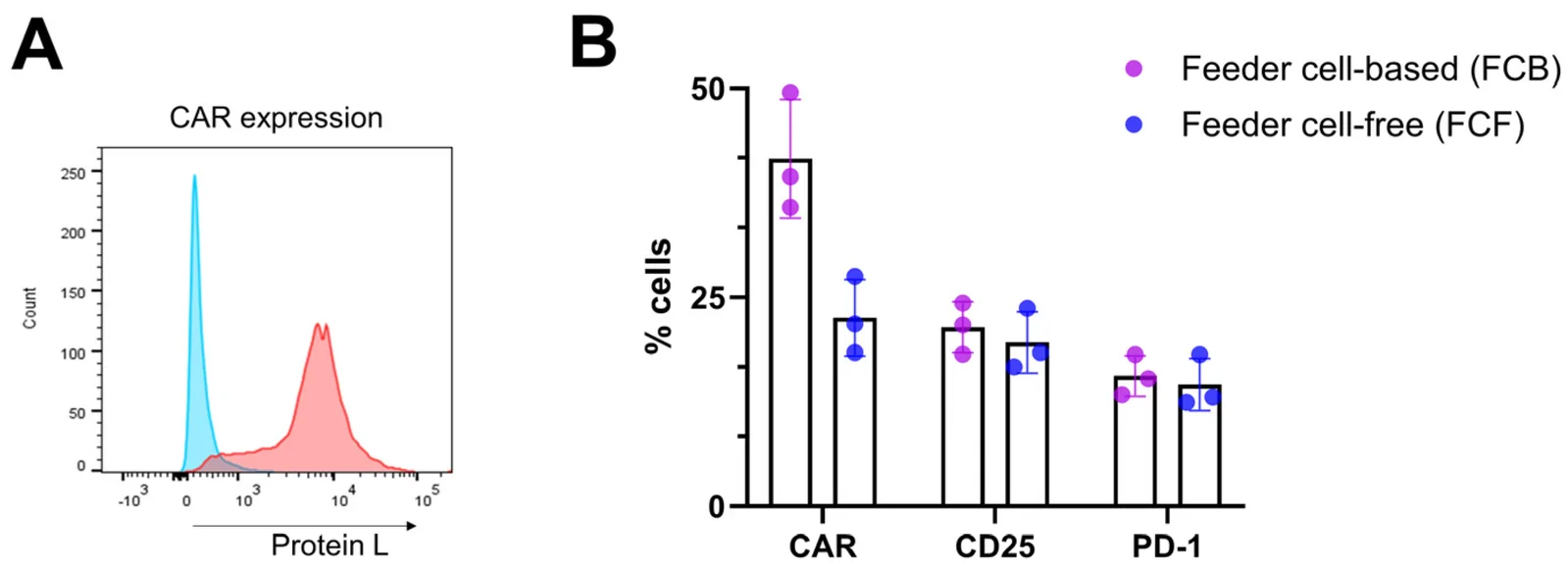
Expression of CAR, CD25, and PD-1 after lentiviral vector transduction: (**A**) Representative histogram demonstrating CAR expression in NK cells 48 h after lentiviral vector transduction (blue: Untransduced, red: Transduced). (**B**) Quantification of CAR, CD25, and PD-1 expression in NK cells after activation using the FCB or FCF method 48 h after lentiviral vector transduction. Each dot represents a donor.

NK cells isolated from PBMCs were transduced with a lentiviral vector. Following 48 h of incubation at 37 °C, CAR expression was measured by flow cytometry using a FITC-conjugated Protein L reagent (Figure 4A). NK cells activated by co-culture with K562 feeder cells (feeder-cell-based (FCB) method) or by PMA/Ionomycin stimulation (feeder-cell-free (FCF) method) were assessed for CAR expression, as well as activation (CD25) and exhaustion (PD-1) markers, following 48 h of lentiviral transduction. The FCB method of NK cell activation resulted in higher levels of CAR expression compared with the FCF method. However, CD25 and PD-1 expression levels were comparable between the FCB and FCF methods (Figure 4B).

### 4.4. CAR-NK Cell Expansion

**Figure 5:**
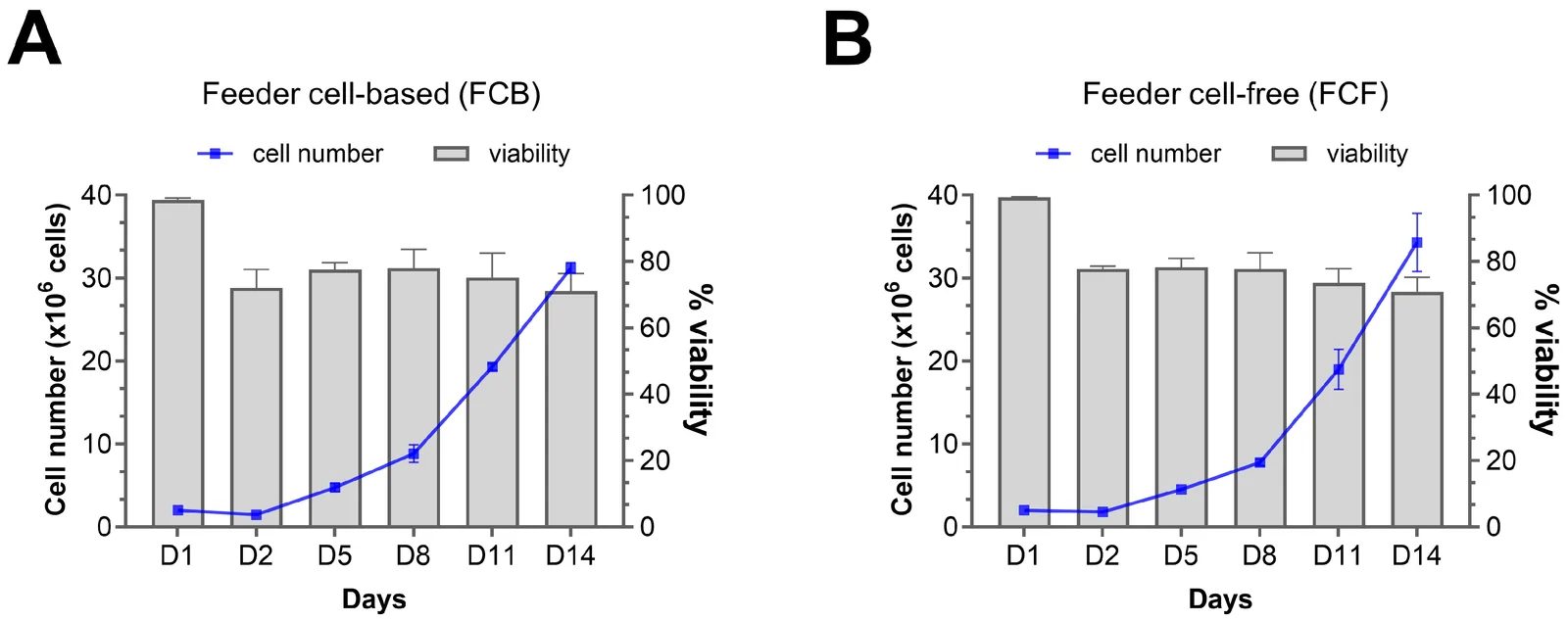
Ex vivo expansion of CAR-NK cells: (**A**) NK cells activated using the FCB or FCF method were expanded after CAR expression by lentiviral vector-mediated transduction. Cell count and viability were measured at various time points during ex vivo expansion using both the (**A**) FCB and (**B**) FCF methods.

NK cells activated using the FCB or FCF method were expanded ex vivo for 14 days. Cell count and viability were measured at various time points. CAR-NK cells expanded by more than 15-fold with both the FCB and FCF methods, and viability remained above 70% at all time points (Figure 5A,B).

### 4.5. Final CAR-NK Cell Product

**Figure 6:**
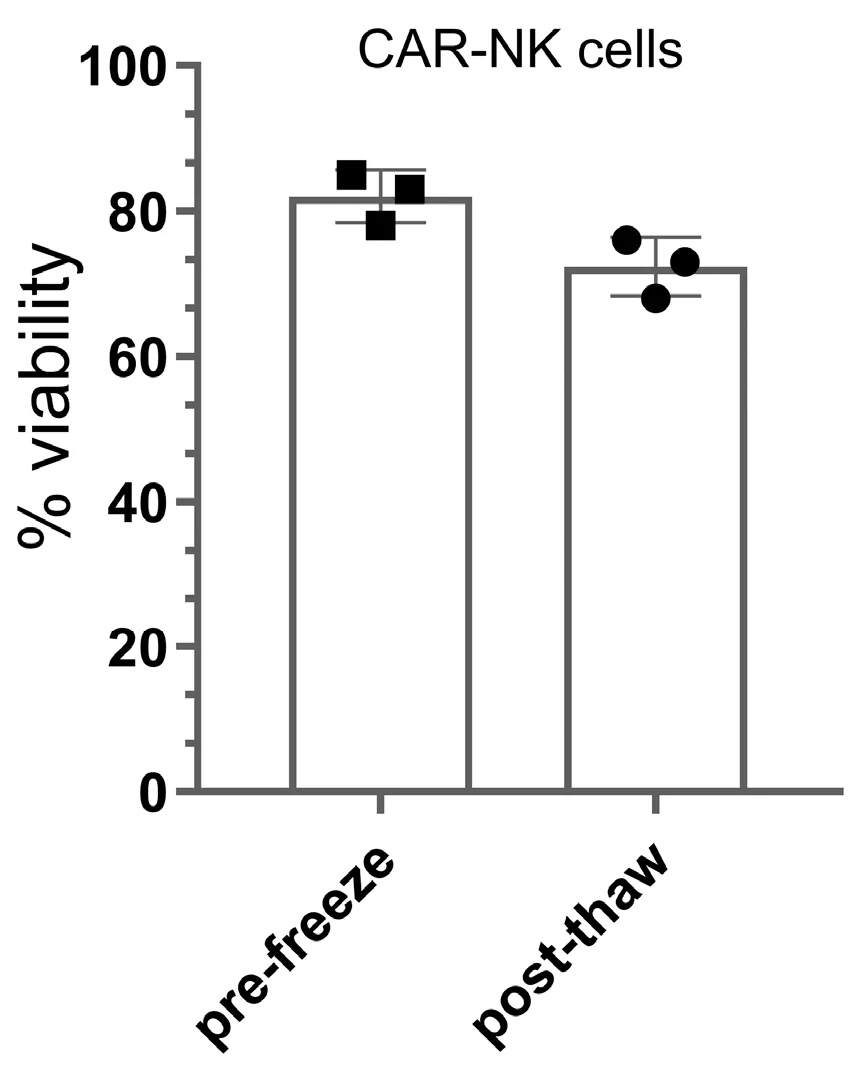
CAR-NK cell viability: CAR-NK cell viability was assessed before cryopreservation (pre-freeze) and after thawing (post-thaw). Each dot represents a donor.

After 14 days of expansion, CAR-NK cells were harvested, washed, formulated in cryopreservation media, and stored at –80 °C for 24 h. Cell viability was measured before cryopreservation (pre-freeze) and after thawing (post-thaw) following 24 h of storage. There was no significant difference in CAR-NK cell viability between the pre-freeze and post-thaw samples (Figure 6).

## 5. Recommendations and Operational Notes

We identify key steps in the CAR-NK manufacturing process—such as transduction efficiency, activation status, expansion kinetics, and cryopreservation—that critically influence product quality and efficacy (Table 2). To mitigate variability and enhance standardization, we recommend certain functional validation assays and define threshold criteria for essential quality attributes: transduction efficiency, cell viability, and CD56^+^/CD3^−^ population purity. These recommendations aim to support harmonization across manufacturing platforms and facilitate reproducible generation of potent CAR-NK cell products, ensuring consistent outcomes across diverse laboratory settings and user experience levels.

### 5.1. Functional Assays

#### 5.1.1. Cytotoxicity Measurement

Most researchers routinely perform 4 h chromium-51 release assays, flow cytometry-based killing assays, or luciferase-based cytotoxicity assays using both CAR antigen-positive and -negative target cell lines. Our standard protocol employs multiple effector–target ratios (1:1, 5:1, 10:1, 20:1) to establish dose–response curves. Depending upon the donor differences, the representative data from anti-CD19 CAR-NK cells demonstrated 25–65% specific lysis against Nalm6 cells at a 5:1 E:T ratio [15].

#### 5.1.2. Degranulation Analysis (CD107a)

CD107a surface expression serves as a marker of NK cells’ functional activity. Generally, we co-culture CAR-NK cells with target cells at multiple effector–target ratios (or PMA/Ionomycin stimulation) for 2–4 h. Functional CAR-NK cells typically show 20–80% CD107a positivity upon antigen encounter, compared with <10% baseline degranulation [6,14,26].

#### 5.1.3. Cytokine Secretion Profiling

We assess IFN-γ, GM-CSF, and TNF-α production through ELISA-based quantification of co-culture supernatants at 4 and 24 h. CAR-NK cells are co-cultured with target cells at multiple effector–target ratios (or PMA/Ionomycin stimulation). Our data consistently show 5–50-fold increases in IFN-γ secretion (typically 100–2000 pg/mL) when CAR-NK cells encounter antigen-positive targets [15].

### 5.2. Donor Variability

We observed significant donor-to-donor variability in transduction efficiency, with rates ranging from 25–65% across different NK cell preparations at an MOI of 20. This variability aligns with published reports demonstrating donor-specific differences in NK cells’ susceptibility to viral transduction [27,28,29,30]. Below are the multiple factors influencing variability among donors and recommendations for MOI and cytokine optimization and usage.

- Starting NK cell purity: Samples with higher CD56^+^CD3^−^ purity (>90%) demonstrated higher and more consistent CAR-NK product attributes.
- Age: NK cells from donors (age < 40 years) typically showed higher transduction and expansion compared with donors (age > 40), consistent with age-related changes in NK cell activation state and cytotoxicity profiles.
- Metabolic state of the NK cell population: Donors with higher baseline NK cell activity showed improved transduction.

#### 5.2.1. Vector Dose Optimization

- Low responders (<20% transduction at MOI 10): Increase the MOI to more than 20 while monitoring cytotoxicity.
- High responders (>80% transduction at MOI 5): Consider reducing to MOI 5 or below to minimize vector-induced toxicity.

#### 5.2.2. Cytokine Supplementation

- IL-15: Increase from the standard 10 ng/mL to 15–20 ng/mL for donors demonstrating poor expansion.
- IL-2: Add fresh IL-2 200 IU/mL on Days 3–5 to improve expansion.
- IL-21: Add 25 ng/mL of IL-21 during G-Rex expansion to improve cellular expansion.

### 5.3. Cell Viability and CAR Expression

We have established time-point-specific viability standards and systematic troubleshooting protocols based on multiple CAR-NK manufacturing runs. Specifically, we define viability acceptance criteria at key checkpoints—post-isolation, post-transduction, and pre-cryopreservation—to ensure cellular integrity and therapeutic efficacy. We have the following recommendations for improving the cell number, viability, and CAR expression.

Viability Acceptance Criteria

- Day 0 (post-isolation): ≥95% viable cells;
- Day 1 (post-transduction): ≥90% viable cells;
- Day 3–7 (early expansion): ≥80% viable cells;
- Day 8–14 (late expansion): ≥70% viable cells;
- Post-cryopreservation: ≥60% viable cells (post-thaw).

#### 5.3.1. Lower Viability During Expansion

- Early stage (Days 3–5, <90%): Check contamination, use fresh media, and maintain cell density (around 1 × 10^6^ cells/mL).
- Mid-stage (Days 7–10, <85%): Assess exhaustion markers and supplement metabolites (2 mM glutamine and 10 mM glucose).
- Late stage (Days 11–14, <80%): Continue fresh cytokine supplementation, maintain cell density, or consider early harvest or cryopreservation.

#### 5.3.2. Poor Cell Expansion (<10-Fold by Day 14)

- Cytokine boost: Increase cytokine concentrations, namely IL-15 to 20 ng/mL and/or IL-21 (15–25 ng/mL) post-transduction.
- Feeder cell co-culture: Optimize the effector–target ratios using irradiated K562-mbIL21 cells if expansion is <5-fold by Day 7.
- Metabolic enhancement: Supplement with 2 mM glutamine and 10 mM glucose.

#### 5.3.3. Low CAR Expression (<20% CAR+ Cells)

- MOI optimization: Increase MOI gradually from 10 to 15–20, monitoring for any vector-related toxicity.
- Transduction enhancement: Use 8 μg/mL polybrene or retronectin coating.
- Timing adjustment: Perform transduction 18–24 h post-activation.

## 6. Discussion

Ex vivo manufacturing of CAR-NK cells begins with the depletion of CD3^+^ cells and enrichment of CD56^+^ NK cells from human peripheral blood mononuclear cells (PBMCs). The purified NK cells are then genetically modified using a lentiviral vector to express the desired chimeric antigen receptor. The resulting CAR-NK cells can be used in various studies to assess their safety, quality, and potency. The manufacturing process is complex and spans multiple days. The quality, safety, and potency of the final product depend on multiple factors, making batch-to-batch variability one of the major challenges in CAR-NK cell manufacturing.

Numerous factors contribute to this variability, including donor-related, process-related, and product-related sources [27,31,32,33,34,35,36]. Donor-related factors such as heterogeneity in the starting material, including donor age, health status, and immune status, play a significant role. Product-related factors, such as heterogeneity in cell phenotype, memory subsets, activation state, and exhaustion, can also significantly impact batch-to-batch consistency. Process-related factors include variability in cytokines, feeder cells, and culture conditions, as well as differences in transduction efficiency, integration sites, manufacturing systems (open vs. closed), production scale, and operator-dependent steps.

Various systems are used to manufacture CAR-NK cells (e.g., small-scale production using traditional cell culture flasks and large-scale production using the G-Rex system). Since CAR-NK cell product attributes may be affected by the manufacturing system, a recent study performed a head-to-head comparison of CAR-NK cells manufactured using the traditional cell culture system and the G-Rex system [15]. Most product attributes were comparable between CAR-NK cells produced using the feeder-cell-based (FCB) and feeder-cell-free (FCF) methods. However, some product attributes differed, and the type of cytokines used during manufacturing also affected the product attributes. Together, these studies highlight the need for stringent manufacturing controls to ensure the consistent quality, safety, and potency of CAR-NK cell products.

In general, the manufacturing process from apheresis collection to final product formulation can take about 10–20 days, depending on the specific protocol [34,37]. The protocol described here typically requires 10–14 days to manufacture NK cells after leukapheresis. The cost of manufacturing can vary greatly between autologous and allogeneic sources. One study estimated that the cost of goods (COG) for producing autologous versus allogeneic cell therapies can differ by as much as 21-fold per dose [38]. The primary drivers of higher costs for autologous products are the single-use consumables and the comprehensive quality control testing required for each individual batch.

Given the use of lentiviral vectors and human blood-derived materials, it is essential to incorporate appropriate biosafety precautions into the protocol. All procedures involving lentiviral vectors should be conducted under Biosafety Level 2 (BSL-2) conditions with additional precautions as recommended by institutional biosafety committees. Personnel must be trained in handling biohazardous materials, and all waste should be properly decontaminated and disposed of in accordance with local and federal regulations. Furthermore, the use of human cells as a starting material may require donor screening and full documentation to minimize the risk of infectious disease transmission, particularly for allogeneic products. Including these biosafety and compliance measures will help ensure that the protocol aligns with institutional, local, and federal regulatory requirements.

## 7. Conclusions

Ex vivo manufacturing of CAR-NK cells begins with the depletion of CD3^+^ cells and the enrichment of CD56^+^ NK cells from human PBMCs. The purified NK cells are then genetically modified, generally using a lentiviral vector, to express the desired chimeric antigen receptor (CAR) targeting antigen(s) of choice. The ex vivo manufactured CAR-NK cells can then be evaluated in various preclinical and clinical studies to assess their safety, quality, and efficacy.

In recent years, several early-phase clinical trials have highlighted the promise of CAR-NK cells as a safe and potentially effective immunotherapy [1,11,39]. CAR-NK cells have been well tolerated with little to no inflammatory toxicities such as severe cytokine release syndrome, neurotoxicity, or graft-versus-host disease, which are commonly seen with CAR-T therapies. These studies underscore the therapeutic potential of CAR-NK cells as an “off-the-shelf” alternative to autologous CAR-T cell products.

Ongoing trials are now investigating CAR-NK cells targeting a range of antigens for the treatment of various cancers and other human diseases. Together, these advancements are paving the way for CAR-NK cells to emerge as a flexible and scalable cellular immunotherapy platform with the potential for broad clinical applications.

## Figures and Tables

**Table 1:** Reagents and Equipment.

General Reagents
HEK293 cells, K562 cells (ATCC, Manassas, VA, USA; CCL-243), phosphate-buffered saline (PBS) without calcium and magnesium, conical centrifuge tubes (15 mL, 50 mL, sterile pipettes, Bovine Serum Albumin (BSA), non-tissue culture coated, and tissue culture coated cell culture plates (24-well or 6-well), RPMI, MACS buffer, penicillin–streptomycin, glutamine, Fetal Bovine Serum, human AB serum, 0.4% Trypan Blue solution, Mr. Frosty, DMSO, cryogenic vials
**Special Reagents**
Ficoll–Paque [density: 1.077 g/mL] (Cytiva, Malborough, MA, USA; 17144002)
Human peripheral blood or buffy coat (preferably collected within 24 h)
RBC lysis buffer (Invitrogen, Carlsbad, CA, USA; 00-4333-57)
CD3 microbeads (Miltenyi Biotec, Gaithersburg, MD, USA; 130-097-043)
CD56 microbeads (Miltenyi Biotec, Gaithersburg, MD, USA; 130-050-401)
Lentiviral vector carrying the desired CAR gene
MACS magnetic separator (Miltenyi Biotec, Gaithersburg, MD, USA; 130-042-602) or equivalent
Retronectin (TakaraBio, San Jose, CA, USA, T100A)
NK MACS (Miltenyi Biotec, Gaithersburg, MD, USA; 130-114-429)
Superblock blocking buffer (Thermo Fischer Scientific, Waltham, MA, USA; 37515)
Phorbol 12-myristate 13-acetate (PMA) (SelleckChem, Houston, TX, USA; S7791)
Ionomycin (SelleckChem, Houston, TX, USA; S7074)
Recombinant IL-2 (Miltenyi Biotec, Gaithersburg, MD, USA; 130-095-760)
Recombinant IL-15 ((Miltenyi Biotec, Gaithersburg, MD, USA; 130-095-760)
Recombinant IL-21 ((Miltenyi Biotec, Gaithersburg, MD, USA; 130-095-767)
G-Rex 6-well plate (Wilson Wolf, Saint Paul, MN, USA; P/N 80240M)
CryoStor CS10 (STEMCELL Technologies, Cambridge, MA, USA; 100-1061)
NK cell expansion media: NKMACs media supplemented with IL-2 (200–500 IU/mL), IL-15 (5 ng/mL), and IL-21(25 ng/mL)
Freezing media: 90% FBS + 10% DMSO or CryoStor CS10
**Equipment**
Incubator (37 °C, 5% CO_2_)
Tabletop centrifuge with swinging-bucket rotor
Countess 3 FL automated cell counter (Thermo Fischer Scientific, Waltham, MA, USA; AMQAF2000)
−80 °C Freezer
Liquid nitrogen (LN2) storage
Water bath (37 °C)
Micropipettes (P10, P20, P100, P200, P1000)

**Table 2:** Summary of the key parameters during CAR-NK manufacturing: Some of the expected outcomes of important parameters such as cell density, cytokine supplementation, viability and CAR transduction are reported.

Parameter	Day 0–2	Day 3–7	Day 8–14	Expected Outcomes
Cell Density	1 × 10^6^ cells/mL	0.5–1 × 10^6^ cells/mL	0.3–0.8 × 10^6^ cells/mL	15–30-fold expansion
IL-15 (ng/mL)	5 (optional)	5	5–10	Sustained activation
IL-2 (IU/mL)	200 (optional)	200–500	200–500	Enhanced proliferation
Media Exchange	None	50% every 48h	50% every 48–72h	Fresh nutrient supply
CAR Expression	N/A	25–55%	25–65%	Stable CAR expression
Viability	>95%	>90%	>85%	>60%

## Data Availability

The original contributions presented in this study are included in the article. Further inquiries can be directed to the corresponding author.
